# Hepatoprotective Effects of Radish (*Raphanus sativus* L.) on Acetaminophen-Induced Liver Damage via Inhibiting Oxidative Stress and Apoptosis

**DOI:** 10.3390/nu14235082

**Published:** 2022-11-29

**Authors:** Kyung-A Hwang, YuJin Hwang, Hye-Jeong Hwang, NaYeong Park

**Affiliations:** 1 Department of Agrofood Resources, National Institute of Agricultural Sciences, Rural Development Administration, Wanju-gun 55365, Republic of Korea; 2 Department of Food and Biotechnology, Korea University, Sejong 30019, Republic of Korea

**Keywords:** radish, *Raphanus sativus* L., acetaminophen, antioxidant, anti-apoptosis, hepatoprotective

## Abstract

Alcohol and drug overdoses cause liver diseases such as cirrhosis, hepatitis, and liver cancer globally. In particular, an overdose of acetaminophen (APAP), which is generally used as an analgesic and antipyretic agent, is a major cause of acute hepatitis, and cases of APAP-induced liver damage are steadily increasing. Potential antioxidants may inhibit the generation of free radicals and prevent drug-induced liver damage. Among plant-derived natural materials, radishes (RJ) and turnips (RG) have anti-inflammatory, anticancer, and antioxidant properties due to the presence of functional ingredients, such as glucosinolate and isothiocyanate. Although various functions have been reported, in vivo studies on the antioxidant activity of radishes are insufficient. Therefore, we aim to evaluate the hepatoprotective effects of RG and RJ in APAP-induced liver-damaged mice. RG and RJ extracts markedly improved the histological status, such as inflammation and infiltration, of mice liver tissue, significantly decreased the levels of alanine transaminase, aspartate aminotransferase, and malondialdehyde, and significantly increased the levels of glutathione, superoxide dismutase and catalase in the APAP-induced liver-damaged mice. In addition, RG and RJ extracts significantly increased the expression of Nrf-2 and HO-1, which are antioxidative-related factors, and regulated the BAX and BCL-2, thereby showing anti-apoptosis activity. These results indicated that RG and RJ extracts protected mice against acute liver injury, attributed to a reduction in both oxidative stress and apoptosis. These findings have clinical implications for the use of RG and RJ extracts as potential natural candidates for developing hepatoprotective agents.

## 1. Introduction

As an essential metabolic organ, the liver transports waste from the body and produces bile acids that break down fat and convert chemical drugs into non-toxic forms [1]. The prevalence of acute liver failure has been increasing due to external (virus, drug overdose) or internal (oxidative stress, metabolic disorder) effects, threatening human health worldwide [2]. In particular, the onset of acute liver failure results from drug-induced liver injury (DILI), and most drugs can cause liver damage [3]. When DILI is induced, the mitochondrial respiratory chain is inhibited, reactive oxygen species (ROS) increase, and adenosine triphosphate depletion occurs [4]. For this reason, mitochondrial dysfunction causes cell apoptosis by regulating the expression of proapoptotic proteins B-cell lymphoma protein 2 (BCL-2) and BCL-2-associated X (BAX), along with oxidative stress [5,6]. Among the many drugs that cause DILI, acetaminophen (APAP) is the most studied [7].

APAP is the most used antipyretic and analgesic drug; it is safe and effective when administered at a therapeutic dose [8,9]. However, APAP overdose produces the toxic metabolite N-acetyl-p-benzoquinone imine (NAPQI), which depletes the antioxidant glutathione (GSH) and induces oxidative stress via ROS generation, further exacerbating liver damage [10]. Owing to this risk, the number of hospital visits and hospitalizations of patients with APAP-induced acute hepatotoxicity has been steadily increasing yearly. However, there are few treatment options for APAP-induced liver damage, and N-acetylcysteine (NAC) is the only clinically available drug. NAC is usually administered orally or intravenously and has been reported safe even at high doses [11]. However, various side effects have been reported in cases of overdose, depending on the route of administration. Oral administration causes nausea and vomiting, and intravenous administration causes anaphylactic reactions such as skin rashes and dyspnea [12,13]. To compensate for these shortcomings, many experiments have been conducted on the ability of natural materials, or active compounds isolated from natural materials, to protect against APAP-induced liver toxicity.

The radish (*Raphanus sativus* L.) is a representative root vegetable of the family *Brassicaceae*, which is extensively cultivated worldwide. Recently, the production of radishes in Korea has reached approximately 1,168,000 tons per year [14]; more than 30% are grown in Jeju, and the cultivated area is continuously increasing. Radishes are an agricultural product with excellent nutritional value owing to their rich moisture and fiber content, and high content of vitamins A and C. In addition, they have traditionally been known to have various benefits, such as digestion-promoting, stomach-protecting, anti-inflammatory, anticancer, antioxidant, and hemostatic benefits [15]. These physiological effects can be observed in various functional ingredients such as glucosinolates [16], isothiocyanates, flavonoids, anthocyanins, alkaloids, saponins, and phenolic acids, which are abundant in radishes [17].

Turnips (*Brassica rapa* L.) have red roots, unlike radishes, and represent a local specialty in Korea, grown on the Ganghwa Island. Turnips contain not only various functional ingredients contained in radishes, but also a large number of antioxidants such as anthocyanins [18,19].

As such, studies on the various physiological activities of radishes and turnips with numerous functional ingredients are being conducted, but studies on the hepatoprotective effects and mechanism of radishes or turnips, and relevant comparative studies, are still insufficient.

As reported by Parikh et al. [20], phenolic compounds of Indian mustard extract, quercetin and catechins, decreased ROS levels, alanine transaminase (ALT) and aspartate aminotransferase (AST) levels in APAP-induced HepG2 cells, thus the hepatoprotective effect of the Brassicaceae’s functional ingredients was confirmed. Based on the study of hepatoprotective effects of Indian mustard extract, a member of the Brassicaceae family, it is also expected that radishes and turnips will have hepatoprotective effects.

Therefore, this study intends to provide basic data for the potential use of radishes and turnips as functional materials by comparing their hepaprotective effects and mechanism on APAP-induced acute liver injuries in mice.

## 2. Materials and Methods

### 2.1. Chemicals

APAP, NP-40, Triton X-100, sodium deoxycholate, sodium chloride, ethylenediaminetetraacetic acid, ethanol, 3-mercaptoethanol, and superoxide dismutase (SOD) quantification kits were purchased from Sigma-Aldrich (St. Louis, MO, USA). Sodium dodecyl sulfate-polyacrylamide gels, polyvinylidene difluoride (PVDF) membranes, and a Chemi-doc image detector (Chemi-Doc XRS+ System) were purchased from Bio-Rad (Hercules, CA, USA). Antibodies against SOD, catalase (CAT), glutathione peroxidase (GPx), nuclear factor erythroid 2-related factor 2 (Nrf2), heme oxygenase-1 (HO-1), BCL-2, BAX, caspase-3, and caspase-9 were purchased from Abcam (Cambridge, MA, USA). The bicinchoninic acid (BCA) protein assay reagent and anti-rabbit immunoglobulin G horseradish peroxidase-conjugated antibodies were purchased from GenDEPOT (Barker, TX, USA). ALT, AST, GSH, and CAT ELISA kits were purchased from Abcam (Cambridge, UK).

### 2.2. Preparation of Sample Extract

The two species of vegetables used in this study; turnips of Ganghwa (RG) and radishes of Jeju (RJ), were purchased from the local markets of Ganghwa and Jeju Islands, South Korea, respectively. We used only the roots in the experiment. The RG and RJ roots were washed thoroughly with distilled water, dried using hot air at 60 °C for 24 h (DS-240BC, DooSung Co., Ltd., Gwangju, Korea), and pulverized using a grinder (IKA, M20, IKA, Staufen, Germany) to prepare the extract. Extraction was performed by adding 70 % ethanol 20 times to the crushed RG and RJ (2.5 kg), stirring at room temperature for 24 h (SMHS-6, DAIHAN Co., Wonju, Korea), and then adding equal volume of solvent as was used in the first extraction to the remaining amount of the solute. The extract was filtered through filter paper (No. 1, Whatman International Ltd., Maidstone, UK), concentrated using a rotary evaporator (EYELA CCA-1110, Rikakikai Co., Tokyo, Japan), and freeze-dried. The freeze-dried sample extract was stored at −20 °C until further use. The yields of RG and RJ extracts were 12% and 26%, respectively.

### 2.3. Animal Experiments

Experiments were performed using BALB/c mice (male, 6-week-old) weighing 20–24 g, purchased from Central Lab Animal Inc. (Seoul, Korea). The animals were retained in a temperature-controlled facility (22 ± 2 °C) and a 12 h light-dark cycle. The mice were allowed ad libitum access to food and water during the experimental period. The use of experimental animals was approved by the Institutional Animal Care and Use Committee of the National Institute of Agricultural Sciences (NAS-202204).

After a week of acclimatization, all mice were randomly divided into the following seven groups (*n* = 7 per group): (1) normal, no sensitization with phosphate buffered saline (PBS); (2) control (CTRL), APAP + PBS; (3) NAC, APAP + 75 mg/kg NAC; (4) RG low (RG 500), APAP + 500 mg/kg RG; (5) RG high (RG 1000), APAP + 1000 mg/kg RG; (6) RJ low (RJ 500), APAP + 500 mg/kg RJ; (7) RJ high (RJ 1000), APAP + 1000 mg/kg RJ. The study design is shown in Table 1. The samples were orally administered (p.o.) to the mice once daily for 4 weeks. The dose of NAC used in the experiment was established according to the method of Saba et al. [21]. The food intake and body weight of the mice were measured weekly. Food intake was calculated as the amount of food consumed per week and the remaining amount from the feed provided.

After 4 weeks, to induce acute liver injury, mice were intraperitoneally injected with 500 mg/kg body weight of APAP, or an equal volume of PBS, followed by starvation for 24 h. At the end of the treatment period, all mice were anesthetized using CO_2_ inhalation and sacrificed. Blood was centrifuged to separate the plasma for biochemical analyses. The liver, spleen, kidney, heart, and colon were dissected, rinsed with PBS, and weighed. Part of the liver tissue was fixed with formalin for histological analysis, and the remaining was immediately frozen at −80°C until further use.

### 2.4. Measurement of Plasma Aminotransferase Levels

Liver injury was evaluated by measuring biomarkers, such as serum ALT and AST levels, using commercial assay kits. Antioxidant enzyme production from plasma was evaluated using commercial kits.

### 2.5. Measurement of Antioxidant Enzymes and Lipid Peroxidation in Plasma

The concentrations of GSH, antioxidant enzymes, such as SOD, CAT, and the lipid peroxide marker malondialdehyde (MDA) were measured using an ELISA kit. All kits were used according to the manufacturer’s instructions.

### 2.6. Histopathologic Examination

For histological analyses, liver samples (*n* = 7 per group) were fixed in 4% formalin, dehydrated, paraffin-embedded, and sectioned into 5 μm slices. The tissues were then stained with hematoxylin and eosin (H&E). Pathological changes in the tissues were observed at 200 × magnification using a camera attached to a microscope (Eclipse TE200, Nikon, Tokyo, Japan). Congestion and infiltration of liver tissue were observed in more than 10 fields of 7 different tissue sections. All histopathologic analyses were performed by an experimenter blinded to the treatment group.

### 2.7. RNA Isolation and Real-Time Polymerase Chain Reaction Analysis

Total RNA from animal liver tissue was isolated using a RNeasy Plus Mini Kit (Qiagen, Valencia, CA, USA). cDNA was then synthesized using M-MLV reverse transcriptase (Promega, Madison, WI, USA), according to the manufacturer’s protocol. The target gene expression level of the synthesized cDNA was measured using the amfiSure qGreen Q-PCR Master Mix (GenDEPOT), and a real-time polymerase chain reaction machine. For relative quantification, mRNA expression was normalized to the levels of glyceraldehyde 3-phosphate dehydrogenase. The primers used are listed in Table 2.

### 2.8. Western Blot Analysis

Liver tissues were lysed using radio immune precipitation assay buffer on ice, and centrifuged at 12,000× *g* for 20 min at 4 °C, for protein extraction. Proteins were quantified using the BCA protein assay. Ten micrograms of protein were separated using 4–20% sodium dodecyl sulfate-polyacrylamide gel electrophoresis (SDS-PAGE) and transferred to PVDF membranes. The transferred membranes were blocked using 5 % (*w*/*v*) skim milk in Tris-buffered saline with 0.1% Tween^®^ 20 Detergent (1 h at room temperature, followed by overnight incubation at 4 °C with β-actin, SOD, CAT, GPx, Nrf2, HO-1, BCL-2, BAX, cleave-Caspase-3 (c-Casp3), and cleave-Caspase-9 (c-Casp9). Subsequently, the membranes were washed and incubated with the secondary antibodies for 1 h at room temperature. The bands were visualized using an enhanced chemiluminescence reagent (Thermo Fisher Scientific, Rockford, IL, USA), and images were captured using a ChemiDoc image detector.

### 2.9. Statistical Analyses

Statistical analyses were performed using SPSS version v25.0 (SPSS, Chicago, IL, USA). Data are expressed as the mean ± standard error (SE) and were analyzed using one-way analysis of variance (ANOVA). Following this, the data were analyzed post hoc using Duncan’s multiple range test and marked with different letters. Statistical significance was set at *p* < 0.05.

## 3. Results

### 3.1. Effects of Radish Extracts on Body Weight, Tissue Weight, and Food Intake of APAP-Induced Liver-Damaged Mice

**Figure 1:**
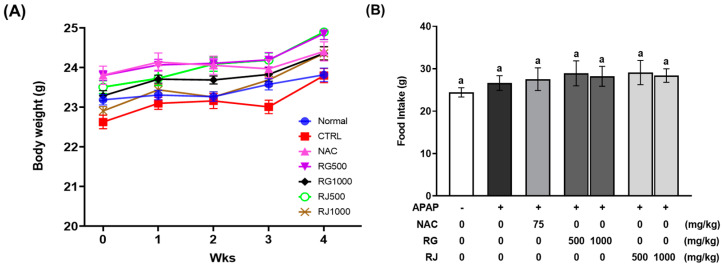
Effects of radish extracts on body weight and food intake in mice. The (**A**) body weight and (**B**) average of food intake for 4 weeks. Results are presented as means ± SE. Different letters represent groups that are significantly different from each other using ANOVA at *p* < 0.05 (*n* = 7). CTRL, control; NAC, N-acetyl cysteine; RG, turnips of Ganghwa; RJ, radishes of Jeju.

To determine the effectiveness of RG and RJ administration, the intake by experimental animals was set to 500 and 1000 mg/kg, respectively, and clinical symptoms were observed during oral administration every day for 4 weeks. There was no significant difference in the body weight and average food intake of the mice for 4 weeks between the CTRL, RG and RJ administered groups (Figure 1 and Figure S1), and no specific symptoms such as odd behavior, ocular protrusions, and hair loss were observed in any of the test groups. In addition, the body weight gain and tissue (liver, spleen, kidney, heart, and colon) weights of mice showed no significant difference between the groups (Table 3).

### 3.2. Radish Extracts Attenuated the APAP-Induced Liver Damage in Mice

**Figure 2:**
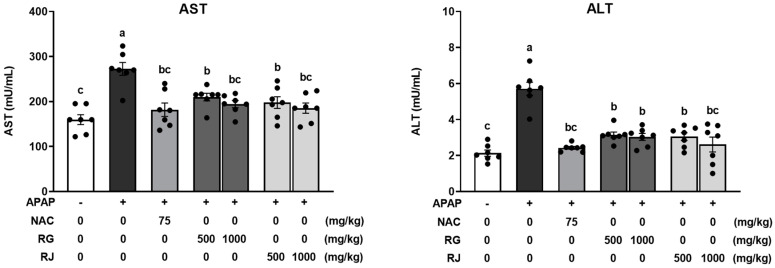
Effects of radish extracts on plasma alanine aminotransferase (ALT) and aspartate transaminase (AST) activity in APAP-induced liver-damaged mice. Results are presented as means ± SE. Different letters represent groups that are significantly different from each other using ANOVA at *p* < 0.05 (*n* = 7). Each dot indicates data measurement for one mouse. CTRL, control; NAC, N-acetyl cysteine; RG, turnips of Ganghwa; RJ, radishes of Jeju.

Liver damage induced by APAP overdose can alter the activities of serum liver enzymes, such as ALT and AST, which are used as indicators of hepatotoxicity [22]. Therefore, plasma ALT and AST levels were used to assess the protective effects of RG and RJ in APAP-induced liver-damaged mice. Plasma ALT and AST levels were significantly (*p* < 0.05) increased in the CTRL group, wherein oxidative stress was induced by APAP. In contrast, the NAC group showed significantly (*p* < 0.05) reduced ALT and AST levels. Compared with the CTRL group, the groups pretreated with RG and RJ showed significantly (*p* < 0.05) decreased ALT and AST levels. In particular, the high concentration radish extract treatment group showed ALT and AST levels similar to the NAC group in the normal range. When only RG and RJ were ingested, the hepatotoxicity index showed no significant difference compared to the normal group (Figure S2). Therefore, RG and RJ alleviated APAP-induced hepatotoxicity (Figure 2).

### 3.3. Effect of Radish Extracts on Hepatotoxicity in APAP-Induced Liver-Damaged Mice

**Figure 3:**
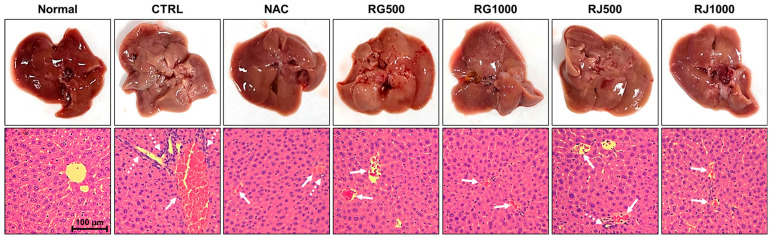
Effects of radish extracts on hepatic histology (hematoxylin and eosin staining) in APAP-induced liver-damaged mice. 200 × magnification. Congestion of central vein (white arrow), neutrophils infiltration (white dotted arrow). (*n* = 7) CTRL, control; NAC, N-acetyl cysteine; RG, turnips of Ganghwa; RJ, radishes of Jeju.

These results were further confirmed using histopathological analysis (Figure 3). In the liver tissue of the normal group, the status of the hepatocyte cells was normal, and no damage was observed. In contrast, in the CTRL group, which was administered only with APAP, significant injury to the liver structure, such as congestion of the central vein and neutrophils infiltration, was observed. Conversely, in the NAC group, the size and degree of infiltration of inflammatory cells decreased and liver tissue injury recovered. In addition, in the experimental group administered with APAP and radish extracts, inflammatory cell infiltration and congestion were reduced in a concentration-dependent manner, improving liver tissue injury. In particular, in the RG 1000 and RJ 1000 group, congestion of the central vein decreased to a similar degree as that in the NAC group, and infiltration of inflammatory cells was significantly reduced compared to that in the CTRL group. Therefore, it was confirmed that RG and RJ extracts alleviated liver damage.

### 3.4. Effects of Radish Extracts on Antioxidant Enzymes and Glutathione Production in APAP-Induced Liver-Damaged Mice

**Figure 4:**
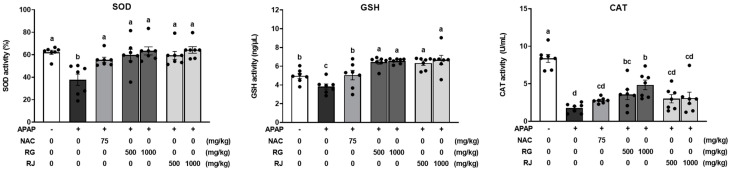
Effects of radish extracts on plasma antioxidant enzymes and glutathione production in the APAP-induced liver-damaged mice. Superoxide dismutase (SOD), glutathione (GSH), and catalase (CAT) levels were measured by ELISA kit. Results are presented as means ± SE. Different letters represent groups significantly different from each other using ANOVA at *p* < 0.05 (*n* = 7). Each dot indicates data measurement for one mouse. CTRL, control; NAC, N-acetyl cysteine; RG, turnips of Ganghwa; RJ, radishes of Jeju.

When excessive oxidative stress is generated in the body, the antioxidant system suppresses oxidative stress by generating antioxidant enzymes such as SOD, GPx, and CAT [23]. However, as APAP depletes the GSH and causes liver damage, the depletion of GSH reduces the activity of antioxidant enzymes SOD and CAT, causing oxidative stress [24,25]. Accordingly, we measured the production of GSH and antioxidant enzymes (SOD and CAT) after RG and RJ treatment in APAP-induced mice with liver damage. As shown in Figure 4, significant decreases in GSH, SOD, and CAT levels were evident in the CTRL group compared to those in the normal group, whereas the NAC group recovered GSH, SOD, and CAT levels. Compared with the CTRL group, groups pretreated with RG and RJ had significantly increased SOD and GSH production. Plasma CAT levels were significantly increased in the RG treatment group.

### 3.5. Effects of Radish Extracts on Lipid Peroxidation in APAP-Induced Liver-Damaged Mice

**Figure 5:**
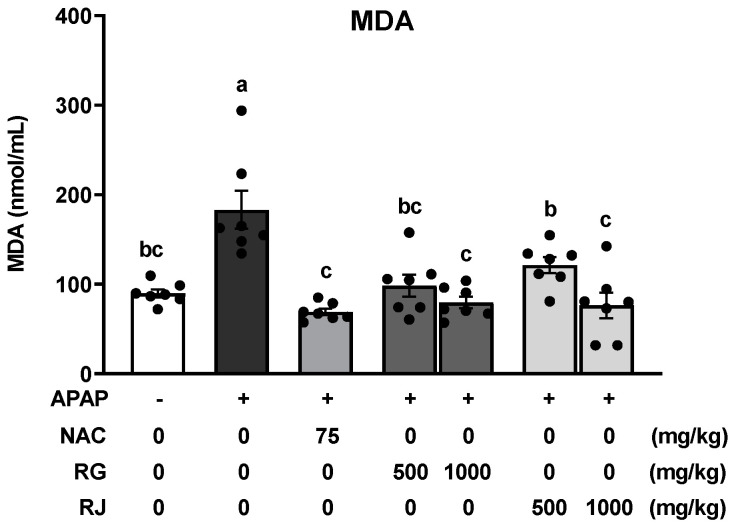
Effects of radish extracts on plasma lipid peroxidase activity in the APAP-induced liver-damaged mice. Malondialdehyde (MDA) production was measured using an ELISA kit. Results are presented as means ± SE. Different letters represent groups that are significantly different from each other using ANOVA at *p* < 0.05 (*n* = 7). Each dot indicates data measurement for one mouse. CTRL, control; NAC, N-acetyl cysteine; RG, turnips of Ganghwa; RJ, radishes of Jeju.

Lipid peroxidation increases during excessive oxidative stress, which primarily contributes to the generation of a number of secondary products, including aldehydes such as MDA, causing direct damage to the cell membrane [26]. Thus, the MDA concentration was measured to evaluate the effect of RG and RJ extract treatment on lipid peroxidation (Figure 5). Compared to the normal group, the CTRL group showed dramatically increased MDA levels (*p* < 0.05), whereas significantly decreased MDA levels were observed in the NAC group. In addition, MDA levels were significantly reduced in the RG and RJ groups, compared with those in the CTRL group, and recovered to a level similar to that of the normal group.

### 3.6. Effects of Radish Extracts on the mRNA Expression of Antioxidant Enzymes in APAP-Induced Liver-Damaged Mice

**Figure 6:**
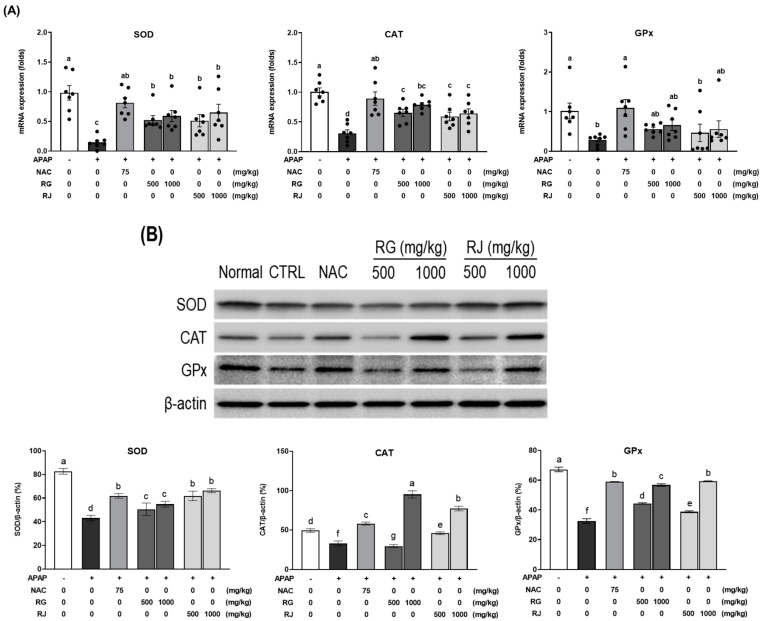
Effects of radish extracts on the mRNA and protein expression of antioxidant enzymes in APAP-induced mice liver tissue. (**A**) mRNA and (**B**) protein expression levels of superoxide dismutase (SOD), catalase (CAT), and glutathione peroxidase (GPx). Results are presented as the means ± SE. Different letters represent groups that are significantly different from each other using ANOVA at *p* < 0.05 (*n* = 7). Each dot indicates data measurement for one mouse. CTRL, control; NAC, N-acetyl cysteine; RG, turnips of Ganghwa; RJ, radishes of Jeju.

The quantification results of plasma antioxidant enzymes confirmed that RG and RJ promoted antioxidant enzyme production in APAP-induced liver-damaged mice. To determine whether a similar tendency is observed in liver tissue as seen in the results of plasma analysis, the protein and mRNA expression of antioxidant enzymes, *Sod*, *Cat*, and *Gpx* were further analyzed (Figure 6). Compared with the normal group, the CTRL group showed dramatically decreased mRNA expression of *Sod*, *Cat* (*p* < 0.05), and *Gpx* (*p* < 0.05), while significantly increased expression levels were exhibited in the NAC group. Decreased *Sod* and *Cat* mRNA expression levels in the CTRL group caused oxidative stress. These levels significantly increased after the oral administration of RG and RJ to APAP-induced liver-damaged mice, and recovered to a level similar to that in the NAC group. The mRNA expression of *Gpx* showed a tendency to increase. In addition, it was confirmed that the protein expression of antioxidant enzymes was decreased by APAP and further increased in a dose-dependent manner after RG and RJ treatment.

### 3.7. Effects of Radishes Extract Regulated Nrf2/HO-1 Signaling Pathway in APAP-Induced Liver-Damaged Mice

**Figure 7:**
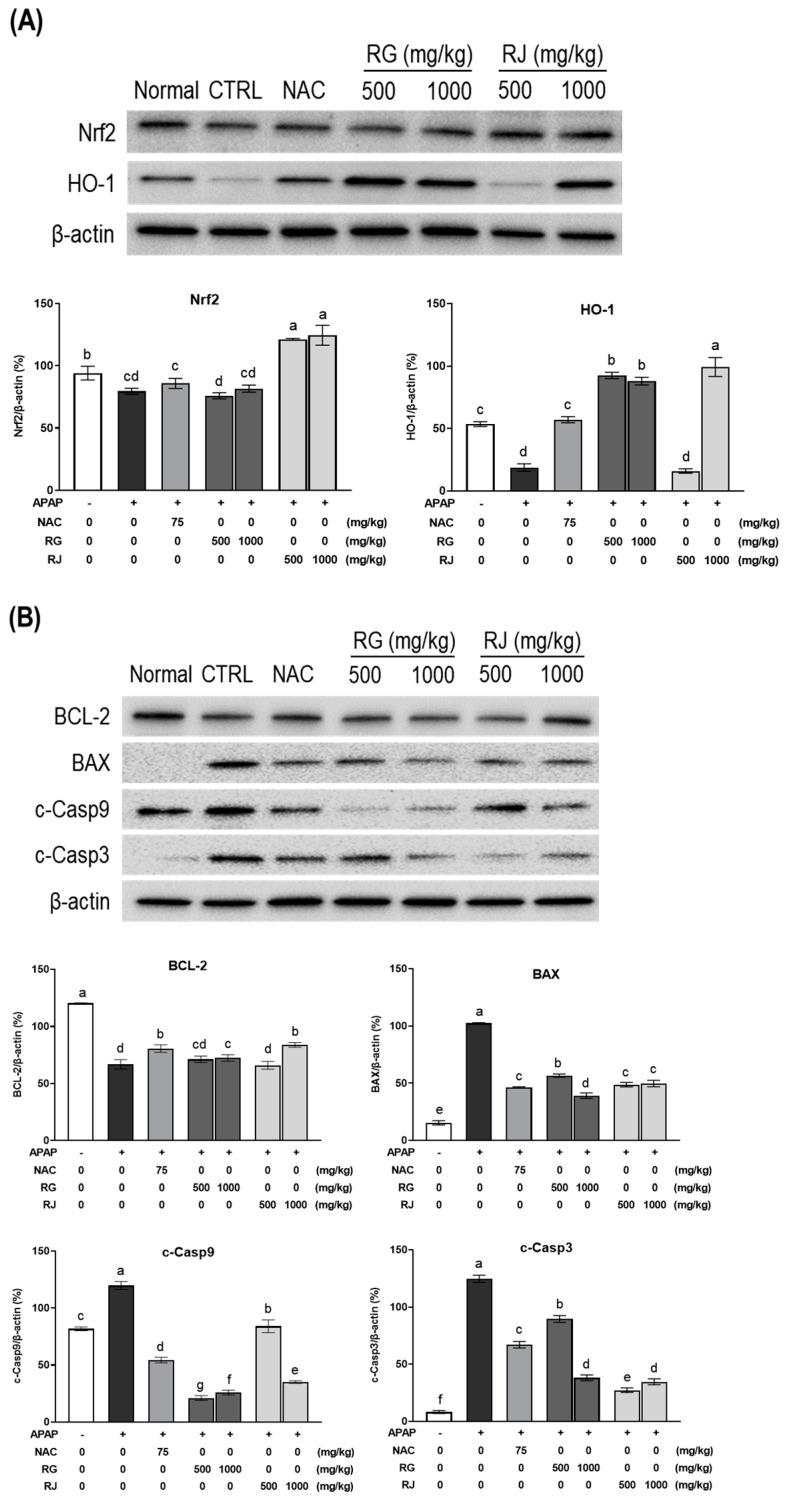
Effects of radish extracts on the protein expression of Nrf2/HO-1 and BCL-2/BAX pathway in APAP-induced liver-damaged mice. Expression levels of (**A**) Nrf2/HO-1 and (**B**) BCL-2/BAX pathway factors including nuclear factor erythroid 2-related factor 2 (Nrf2), heme oxygenase-1 (HO-1), B-cell lymphoma protein 2 (BCL-2), BCL-2-associated X (BAX), Caspase3 (Casp3), and Caspase9 (Casp9). Results are presented as means ± SE. Different letters represent groups that are significantly different from each other using ANOVA at *p* < 0.05. CTRL, control; NAC, N-acetyl cysteine; RG, turnips of Ganghwa; RJ, radishes of Jeju.

To gain further insights into the molecular mechanisms of RG and RJ functions as an antioxidant, the Nrf2/HO-1 mechanism factor was examined using western blotting (Figure 7). Nrf2 forms a complex with Kelch-like ECH-associated protein 1 (Keap1) under normal circumstances and exists in an inactivated state [27] and is a major regulator of the antioxidant defense mechanism of cells [28]. After oxidative stress stimulation occurs, it is separated from Keap1, moves into the nucleus, binds to the antioxidant response element (ARE) site, and expresses HO-1, a signaling downstream factor [29,30]. The expressed HO-1 activates antioxidant enzymes such as SOD, CAT, etc., and activates the antioxidant system.

The expression of Nrf2 significantly increased in the RJ-treated groups compared to the CTRL group. In addition, the HO-1 protein expression was restored by ingestion of RG and RJ at 1000 mg/kg.

Disruption of this oxidative stress-relieving mechanism or excessive ROS generation induces apoptosis. Apoptosis destroys cellular metabolic function owing to DNA damage, and the activity of the pathway leading to this is regulated by BCL-2 and its antagonist, BAX [31]. BCL-2 protein expression was decreased in the oxidative stress-induced group (CTRL) compared to that in the normal group, and the expression of BAX increased, confirming apoptosis. As a result of ingestion of RG and RJ extracts for 4 weeks in mice with an activated apoptotic mechanism, the expression of BCL-2 and BAX were restored to the levels of the NAC group. The apoptosome, which is an early complex protein of apoptosis, is released by regulating the activity of apoptosis factors. The apoptosome converts pro-caspase 9 to its active form, cleaved caspase-9, and further activates the effector caspase-3 and induces cell apoptosis [32,33]. Evaluation of the effect of the extracts on the expression level of these caspases confirmed that apoptosis was inhibited in the RG and RJ groups as opposed to the CTRL group, which promoted apoptosis.

These results indicated that RG and RJ extracts inhibited APAP-induced apoptosis, thereby exhibiting a hepatoprotective effect.

## 4. Discussion

APAP is a general drug that exhibits antipyretic and analgesic effects when taken within the normal range [34]. However, as overdose of the drug causes acute liver failure, the US Food and Drug Administration (FDA) in 2011 limited the daily dose of APAP not to exceed 325 mg, and recommended a daily dose of 4 g [35]. APAP overdose causes acute liver failure because the liver’s rapid metabolic rate makes it vulnerable to drugs and toxins [36]. These APAP-induced hepatotoxicity symptoms are known to be mainly regulated by the mechanisms of inflammation, autophagy, and oxidative stress [37,38,39]. Therefore, developing effective compounds that regulate the primary mechanisms to alleviate and prevent APAP-induced hepatotoxicity is essential.

In this study, the hepatoprotective effect and mechanism of action of RG and RJ, which are natural plant-derived substances, were investigated in APAP-induced liver-damaged mice to test whether they could prevent and alleviate diseases caused by oxidative stress and apoptosis.

First, we measured the production of biochemical markers, such as ALT and AST, which indicate the presence of liver damage. It was confirmed that serum ALT and AST levels increased in the group (CTRL) induced by oxidative stress with APAP and recovered to the normal group levels in the RG and RJ extracts.

Second, when oxidative stress occurs, the antioxidant system protects the tissues in the body, and antioxidants are produced for smooth metabolic activity. SOD converts superoxide radicals (O_2_^•−^) into molecular oxygen (O_2_) and hydrogen peroxide (H_2_O_2_) to prevent cellular damage by ROS [40]. GSH removes excess ROS and plays a vital role in protecting the cells from oxidative stress. In addition, lipid hydrogen peroxide and free hydrogen peroxide produced during oxidative stress are reduced to the corresponding alcohols and water by GPx [41]. Hydrogen peroxide is a harmful by-product of metabolic processes. It must be converted into other less hazardous substances to prevent damage to cells and tissues; thus, CAT needs to be rapidly generated to catalyze its decomposition into gaseous oxygen and water molecules [42]. As such, all factors must play their respective roles for the antioxidant system to work. If any of these factors are depleted or the function is impaired, an error in the antioxidant system leads to cell damage and apoptosis. Overdose of APAP depletes GSH in the liver. GSH depletion inhibits the decomposition of superoxide radicals, which in turn affects SOD activity and ROS removal, leading to acute liver failure [24,25]. As a result, the dysfunction of the antioxidant system occurs as a chain reaction. Therefore, this study estimated the amount of antioxidant enzymes in blood after radish intake in the APAP-induced liver-damaged mice. It was confirmed that the blood levels of GSH, antioxidants SOD and CAT, and lipid peroxide MDA, were regulated by RG and RJ extract treatment, as opposed to the CTRL group. This result is consistent with the finding that APAP-induced acute liver damage decreases antioxidant GSH activity and increases lipid peroxide production [43]. As the regulation of lipid peroxide production is essential for the prevention of liver damage and is regulated by the antioxidant system, RG and RJ suppressed the production of lipid peroxide by increasing the production of antioxidants, thereby demonstrating antioxidative effects [44].

Finally, an overdose of APAP causes liver damage; therefore, a histopathological study was performed. APAP administration externally changed the liver’s color, inflammation, cell infiltration, and central venous congestion as observed via H&E staining. These results confirm that APAP induces liver toxicity, resulting in liver tissue inflammation and cell infiltration [45]. However, the symptoms were relieved in a dose-dependent manner by RG and RJ extracts. Accordingly, RG and RJ proved effective in protecting the liver.

After confirming the hepatoprotective effect of radish extract using liver toxicity indicators such as blood antioxidant enzymes, lipid peroxide, and histopathological analysis, we measured the expression of factors involved in the antioxidant pathway, Nrf2/HO-1 and BCL-2/BAX, to determine the detailed hepatoprotective mechanism of radish and turnip extracts. The expression of antioxidant enzymes SOD, CAT, GPx, and antioxidant system regulator Nrf2/HO-1 in the liver damage-induced group was reduced and reversed by the RG and RJ extracts. In addition, the expression of BCL-2, BAX, c-Caspase3, and c-Caspase9, which are related to apoptosis control pathways, also showed contrasting trends in the RG and RJ groups compared to those of the CTRL group. Our results showed that RG and RJ upregulated the Nfr2 upstream factor and the downstream factor HO-1, thereby increasing antioxidant enzyme activity. In addition, it has been demonstrated that it regulates mitochondrial-dependent apoptosis via Bcl-2 and Bax [46], and inhibits proteolytic activity and apoptosis by inhibiting caspase3 and caspase9 [47].

These results are similar to those previously reported on the association between oxidative stress and apoptosis. Among several antioxidant mechanisms, Nrf2/HO-1 signaling plays a vital role in cell migration, growth, and death [48]. The pathway of Nrf2 is a key sensor that protects cells from oxidative stress by inducing cytoprotective gene transcription and is linked to cytoplasmic Keap1 in steady state. At this time, when ROS is excessively generated by oxidative stress, Keap1 releases Nrf2 and then migrates to the nucleus where it binds to AREs associated with other transcription factors and helper proteins. It induces transcription of several antioxidant genes, such as NAD(P)H:quinone oxidoreductase 1 (NQO1), HO-1, glutamate-cysteine ligasecatalytic subunit (GCLC), CAT, and SOD [49,50]. In addition, there are an increasing number of studies that can support Nrf2, the antioxidant key modulator, as an important factor in improving liver damage [51,52]. It is known that Nrf2 activates HO-1 expression, and upregulates the apoptosis inhibitor BCL-2, and on the contrary, downregulates the expression of BAX, c-Caspase3, and c-Caspase9 [53,54]. Studies indicate that oxidative stress is improved and apoptosis is suppressed by Nrf2, a multi-signaling pathway modulator [55]. Homing et al. [56] confirmed that the hepatoprotective effect of Lico A against acetaminophen-induced hepatotoxicity was via the regulation of Nrf2 and c-Caspase3, and the knockout of hepatocyte-specific JNK can alleviate mitochondrial oxidative stress and reduce apoptosis and liver damage [57]. Therefore, our radish and turnip extracts are also assumed to regulate oxidative stress and apoptosis by activating the Nrf2 factor. This effect may be attributed to glucosinolate, a compound in *Brassicaceae*. Glucosinolate is a sulfur-containing substance mainly found in the *Brassica* family, and has been reported to possess various physiological effects, such as anti-inflammatory and anticancer activities [58,59]. Isothiocyanate, a secondary hydrolysate formed by glucosinolate hydrolysis, is a biologically active compound with anticarcinogenic [60] and antifungal [61] properties. In particular, it regulates the Keap1/Nrf2/ARE pathway and promotes antioxidant and apoptotic effects in cancer cells [58]. Additionally, various functions related to the hydrolysis of glucosinolate metabolites have been reported. Phenethyl isothiocyanate, released when the tissue of *Brassica* vegetables is destroyed, induces apoptosis via the activation of proteins (mitogen-activated protein kinases, c-Jun-N-terminal kinase, extracellular protein kinase, and p38) in colon cancer cells HT29 [62,63]. Sulforaphane (SFN), another glucosinolate hydrolysis product, is used primarily as an enzyme, a cell cycle arrest regulator, an antioxidant, and an anticancer agent [64]. SFN treatment in HepG2 cells increases GSH levels, thus demonstrating antioxidant effects by inhibiting ROS production [65]. In addition to the functional components, the antioxidant and anticancer effects of *Brassica* vegetables are correlated with the presence of natural antioxidant vitamins A, C, and E [59].

Taken together, it was confirmed that in the RG and RJ extract treatment groups, liver damage and antioxidant enzyme expression levels were restored to the levels of the NAC group or the normal group in APAP-induced liver-damaged mice. In particular, the hepatoprotective, antioxidant and anti-apoptotic effects were the greatest at a concentration of 1000 mg/kg of the two extracts, and there was no significant difference in the efficacy between the two extracts. These results are thought to be due to the components commonly included in the two extracts. In order to elucidate the exact mechanism of the hepatoprotective effects of radishes and turnips, research on active components and clinical studies should be conducted in the future. Moreover, the correlation between analysis of ingredients and functionality by harvest period and cultivation area should also be studied.

## 5. Conclusions

This study demonstrated the hepatoprotective effects of RG and RJ by regulating antioxidant and anti-apoptotic signaling factors. An overdose of APAP induces oxidative stress and apoptosis, ultimately resulting in liver damage. As this phenomenon could be successfully alleviated by RG and RJ extracts, further studies on the mechanism can aid in the use of radish and turnip extracts as potential natural candidates for the development of hepatoprotective agents.

## Figures and Tables

**Table 1:** Animal experimental groups and treatments.

Condition	Groups	Treatment (mg/kg)
Non	Normal	PBS
Acute liver injury (APAP, 500 mg/kg)	CTRL	PBS
	NAC	N-acetylcysteine (75)
	RG 500	Turnips of Ganghwa (500)
	RG 1000	Turnips of Ganghwa (1000)
	RJ 500	Radishes of Jeju (500)
	RJ 1000	Radishes of Jeju (1000)

CTRL, control; PBS, phosphate buffered saline; APAP, acetaminophen.

**Table 2:** Primer sequences for Real-Time Polymerase Chain Reaction RT-PCR.

Gene	Primer	Sequences (5′→3′)
*Sod*	Forward	GGGTTGGCTTGGTTTCAATAAGGAA
	Reverse	AGGTAGTAAGCGTGCTCCCACACAT
*Cat*	Forward	AAGACAATGTCACTCAGGTGCGGA
	Reverse	GGCAATGTTCTCACACAGGCGTTT
*Gpx*	Forward	CTCGGTTTCCCGTGCAATCAG
	Reverse	GTGCAGCCAGTAATCACCAAG
*Gapdh*	Forward	GAGCCAAAAGGGTCATCATC
	Reverse	TAAGCAGTTGGTGGTGCAGG

**Table 3:** Tissue weight of APAP-induced *liver*-*damaged* mice.

Treatment		Body Weight Gain (g)	Liver (g)	Spleen (g)	Kidney (g)	Heart (g)	Colon (g)
Normal		0.633 ± 0.479 ^b^	1.049 ± 0.016 ^a^	0.088 ± 0.001 ^ab^	0.357 ± 0.005 ^a^	0.125 ± 0.003 ^ab^	0.205 ± 0.003 ^a^
CTRL		1.171 ± 0.144 ^ab^	0.977 ± 0.017 ^a^	0.089 ± 0.002 ^a^	0.351 ± 0.008 ^a^	0.124 ± 0.002 ^ab^	0.213 ± 0.003 ^a^
NAC		0.600 ± 0.200 ^b^	0.968 ± 0.020 ^a^	0.083 ± 0.002 ^ab^	0.352 ± 0.005 ^a^	0.121 ± 0.001 ^ab^	0.214 ± 0.003 ^a^
RG	500	1.057 ± 0.268 ^ab^	1.033 ± 0.012 ^a^	0.077 ± 0.002 ^ab^	0.367 ± 0.004 ^a^	0.131 ± 0.002 ^a^	0.223 ± 0.003 ^a^
	1000	1.071 ± 0.203 ^ab^	0.971 ± 0.016 ^a^	0.087 ± 0.002 ^a^	0.379 ± 0.005 ^a^	0.128 ± 0.003 ^ab^	0.207 ± 0.003 ^a^
RJ	500	1.386 ± 0.122 ^ab^	0.975 ± 0.009 ^a^	0.088 ± 0.002 ^a^	0.356 ± 0.006 ^a^	0.127 ± 0.002 ^ab^	0.197 ± 0.004 ^a^
	1000	1.433 ± 0.237 ^b^	0.973 ± 0.005 ^a^	0.084 ± 0.001 ^ab^	0.356 ± 0.004 ^a^	0.115 ± 0.001 ^b^	0.197 ± 0.004 ^a^

Data are expressed as mean ± SE. Different letters represent groups that are significantly different from each other according to ANOVA (*p* < 0.05) (*n* = 7). CTRL, control; NAC, N-acetyl cysteine; RG, turnips of Ganghwa; RJ, radishes of Jeju.

## Data Availability

Not applicable.

## Supplementary Materials

The following supporting information can be downloaded at: https://www.mdpi.com/article/10.3390/nu14235082/s1, Figure S1: Effects of radish extracts on body weight percent and daily food intake in mice.; Figure S2: Effects of radish extracts on plasma alanine aminotransferase (ALT) and aspartate transaminase (AST) activity in the mice.
