# Supplementary material for: Hepatoprotective Effects of Radish (Raphanus sativus L.) on Acetaminophen-Induced Liver Damage via Inhibiting Oxidative Stress and Apoptosis
Source: Nutrients. 2022 Nov 29;14(23):5082. doi: 10.3390/nu14235082 (PMC9737327; doi:10.3390/nu14235082)
Supplement: Supplementary file 1 [file nutrients-14-05082-s001.zip › nutrients-1951956-supplementary.pdf]

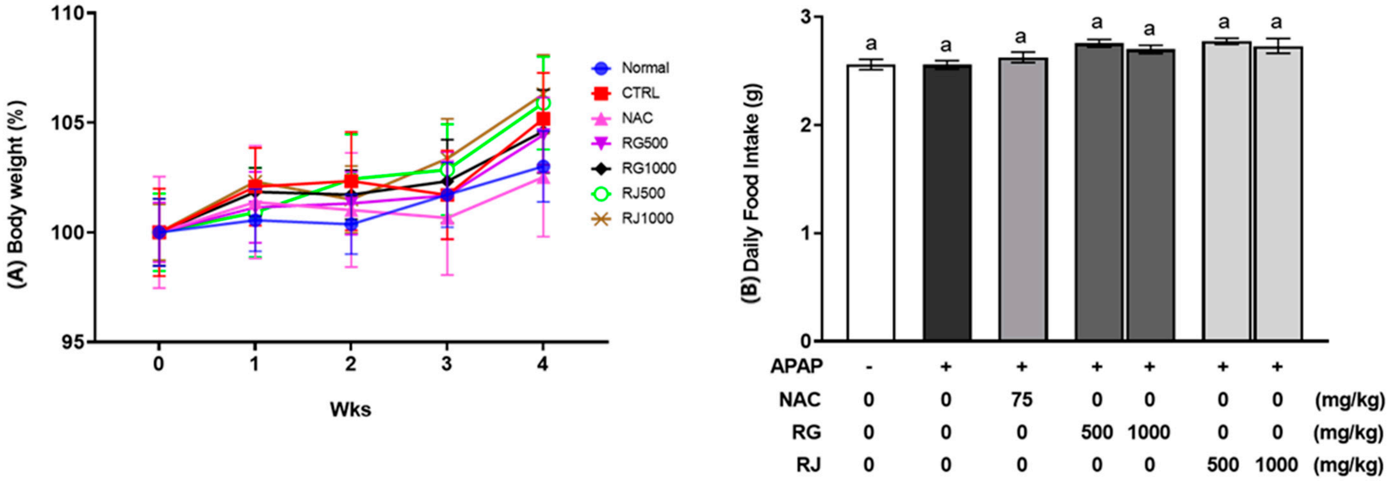

Figure S1. Effects of radish extracts on body weight percent and daily food intake in mice. The (**A**) body weight represented as percent change from 0 wks weight and (**B**) daily food intake of one mouse per group fed a normal diet was measured once a week for four weeks. Results are presented as means ± standard error of mean (SEM). Different letters represent groups that are significantly different from each other using analysis of variance (ANOVA) at $p < 0.05$ (n=7). CTRL, control; NAC, N-acetyl cysteine; RG, radish of Ganghwa; RJ, radish of Jeju; APAP, acetaminophen.

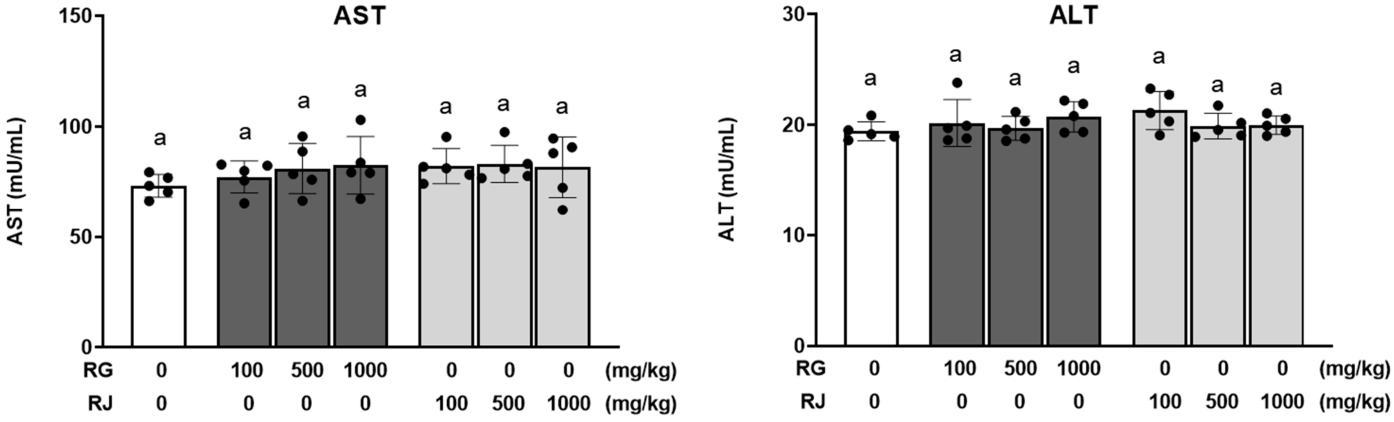

Figure S2. Effects of radish extracts on plasma alanine aminotransferase (ALT) and aspartate transaminase (AST) activity in the mice. Results are presented as means ± SEM. Different letters represent groups that are significantly different from each other using ANOVA at $p < 0.05$ (n=5). Each dot indicates data measurement for one mouse. RG, radish of Ganghwa; RJ, radish of Jeju.
